# Spindle cell carcinoma of the tongue: a rare variant of squamous cell carcinoma

**DOI:** 10.3332/ecancer.2014.447

**Published:** 2014-07-21

**Authors:** Manisha V Biradar, Sunita S Dantkale, Rahul S Abhange, Hemlata T Kamra, Khushboo Birla

**Affiliations:** Department of Pathology, Government Medical College, Latur 413512, India

**Keywords:** biphasic, epithelial, mesenchymal, spindle cell, squamous

## Abstract

Spindle cell carcinoma (SpCC), a rare, aggressive variant of squamous cell carcinoma (SCC), is characterised by proliferation of epithelial and mesenchymal components. It is important to diagnose this variant of SCC, because of its tendency to recur and early metastasis. It accounts for 1% of all tumours in the oral cavity. In this paper, we have reported a case of SpCC of the tongue in a 65-year-old male who presented with a polypoidal growth over the lateral border of his tongue with a short history of one month. Immunohistochemical expression of cytokeratin was strongly positive in the epithelial component and focally in the spindle cell component. The spindle cell component showed a strong positivity for vimentin.

## Introduction

Spindle cell carcinoma (SpCC) of the head and neck is a rare, biphasic neoplasm first described by Virchow in 1865 [1]. It is composed of squamous cell carcinoma (SCC), either *in situ* and/or in invasive form, and a malignant spindle cell component with a mesenchymal appearance, but of epithelial origin [2]. It accounts for 3% of all SCCs in the head and neck region [3]. It is most commonly encountered in the upper aero digestive tract [1, 2], the larynx (particularly the vocal cords) and hypo pharynx being the most common sites and rarely in the oral cavity [4]. The majority of SpCC cases occurs in men (85%), most frequently between the sixth and eighth decades of life [5]. It has been linked to cigarette smoking, alcohol abuse, and previous radiation exposure to the affected area [2–4]. We present a case report of this rare tumour, with an unusual location, to contribute in part to better understanding and awareness of this rare malignancy.

## Case report

A 65-year-old male patient presented with a painless, polypoidal lesion over the tongue, rapidly grown over a period of one month. Physical examination revealed a non-tender, non-ulcerated pedunculated exophytic mass arising from the right lateral border of the tongue. The mass was adherent to underlying structures. The patient did not have a history of pre-existing SCC or radiation therapy, and there was no regional lymphadenopathy. Clinical diagnosis of lingual SCC was made. An excisional biopsy was done and sent for histopathological examination.

**Figure 1.**
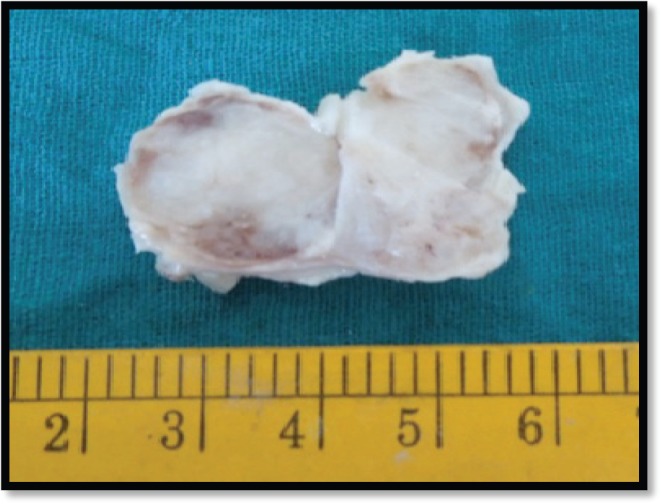
Gross-grey white mass.

**Figure 2.**
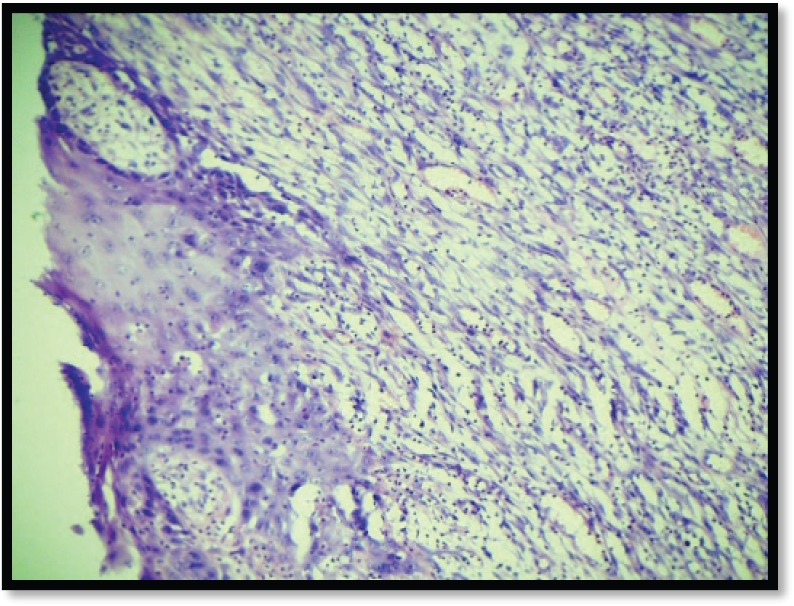
M/E showing squamous and spindle cell component (H & E Stain; 10×).

**Figure 3.**
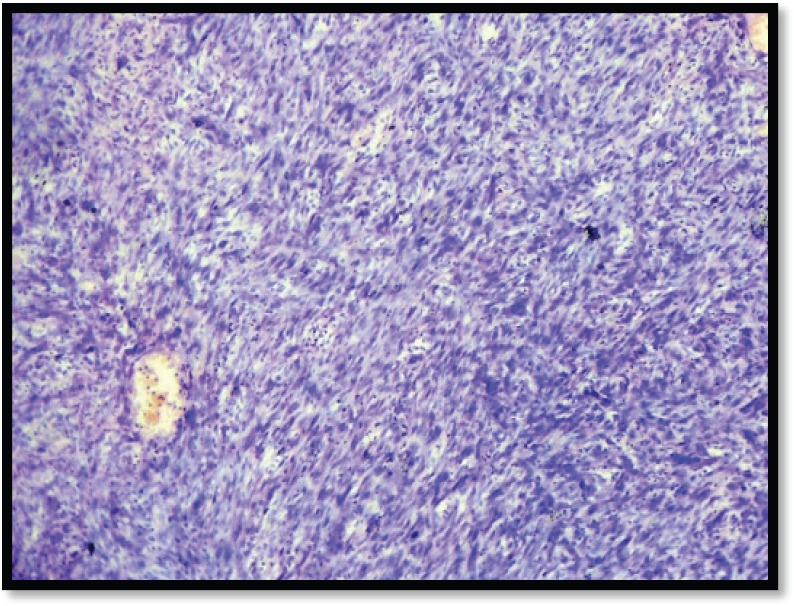
M/E showing spindle cells arranged in fascicles (H & E Stain; 10×).

**Figure 4.**
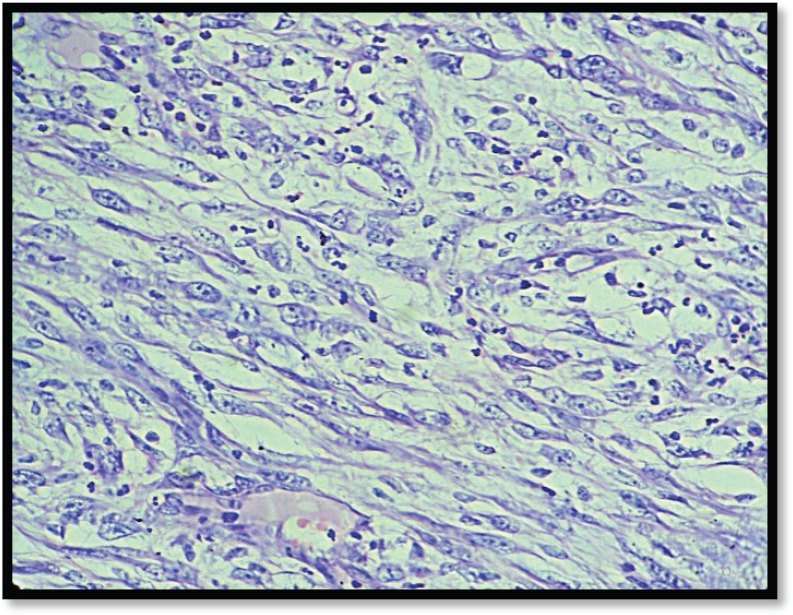
M/E showing spindle cell component (H & E Stain; 40×).

**Figure 5.**
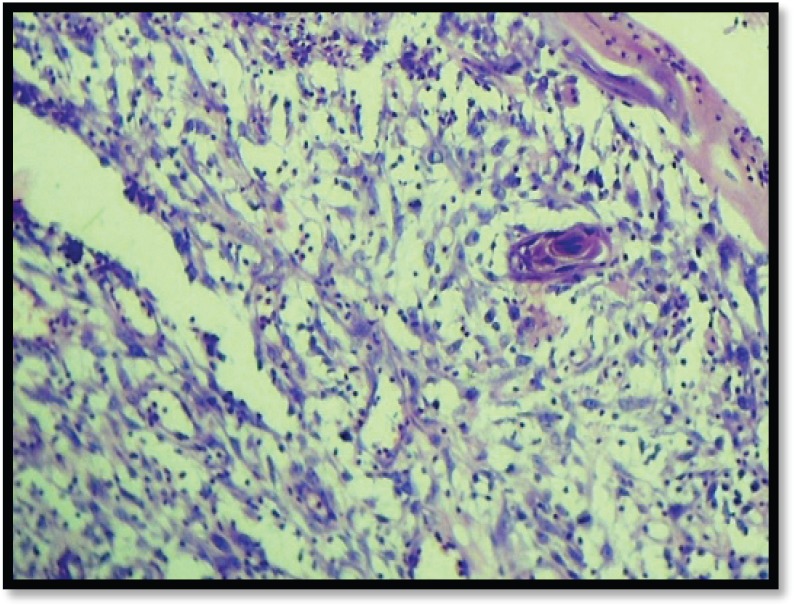
M/E showing epithelial pearl (H & E Stain; 10×).

**Figure 6.**
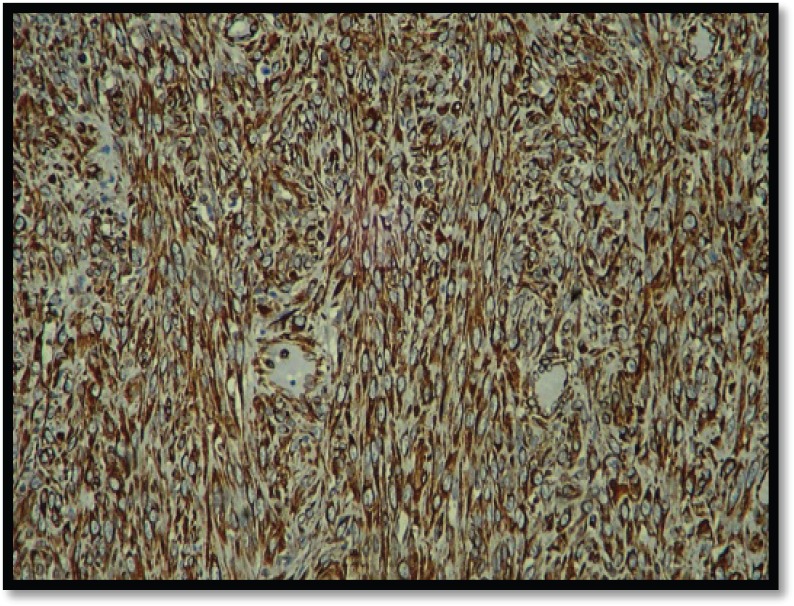
IHC showing spindle cells strongly positive for vimentin (10×).

**Figure 7.**
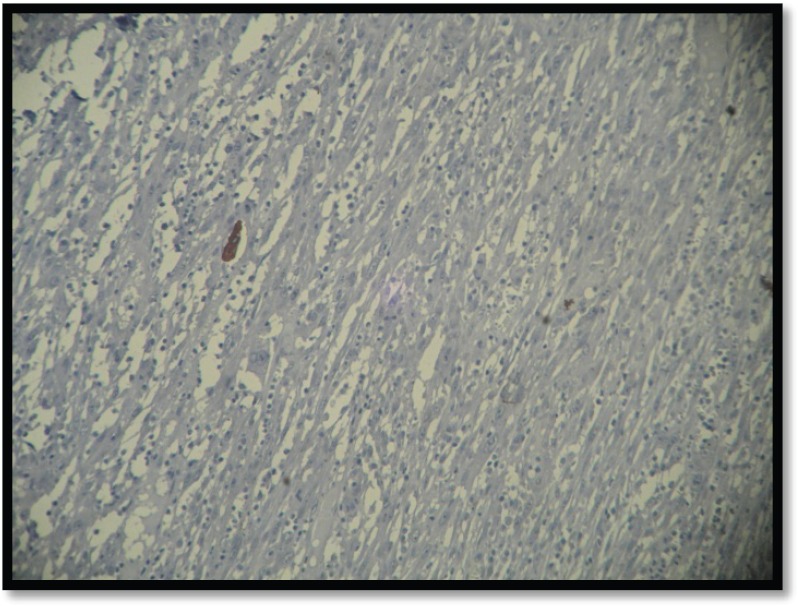
IHC showing spindle cells focal positivity for cytokeratin (10×).

**Figure 8.**
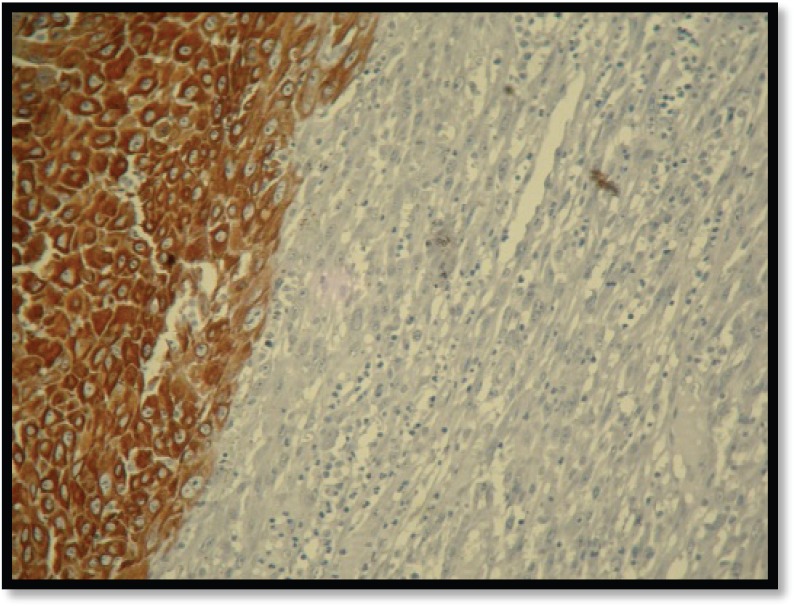
IHC showing squamous cells positive for cytokeratin (10×).

Grossly, the mass was 2 × 2 × 1 cm polypoidal, grey-white, and hard in consistency. The cut surface was grey-white solid homogeneous [Figure 1]. Microscopic examination revealed a tumour composed of proliferating atypical bipolar spindle cells and small nests of squamous epithelial cells [Figure 2]. Malignant spindle cells, arranged in fascicles [Figures 3 and 4], formed the main bulk of the tumour. The cells had increased nuclear-cytoplasmic ratio with round to oval nuclei and prominent nucleoli and a moderate amount of eosinophilic cytoplasm. In between, the spindle cell numerous proliferating capillaries and inflammatory cells were also noted. At a few foci, small nests of malignant squamous epithelial cells and epithelial pearls were seen [Figure 5]. The tumour showed surface ulceration and areas of haemorrhage. The lesion was diagnosed as spindle cell neoplasm. On immunohistochemistry, vimentin was strongly positive in the spindle cell component [Figure 6] and negative in the epithelial component. Cytokeratin was focally positive in spindle cell component [Figure 7] and strongly positive in the epithelial component [Figure 8].

## Discussion

SpCC is an unusual form of poorly differentiated SqCC [6]. These tumours are uncommon in the oral cavity; reportedly accounting for less than 1% of all tumours of the oral regions [2]. Very limited literature is available on the localisation of SpCC on the tongue [4]. Many terms, including pseudo sarcoma, sarcomatoid carcinoma, collision tumour, carcinosarcoma, pleomorphic carcinoma, and polypoid carcinoma have been applied. This reflects the divergent interpretation of the histogenesis of the spindle cell component [6].

SpCC primarily affects men between sixth and seventh decades of life [2–4]. The spindle cell variant is very rare in childhood and adolescence, though Kesseler *et al*. had reported SpCC on the tongue in a 4-year-old boy [7]. SpCC most frequently affects the larynx [3, 4], however, it may rarely occur in various organs: gingiva, tongue, upper aerodigestive tract, including hypopharynx and nasal cavity, oesophagus, skin, and breast [8]. In our case, SpCC was seen arising from the tongue, which is a very rare site. Potential risk factors include the history of tobacco use, poor oral hygiene, alcohol abuse, and previous ionizing irradiation of the area [2–4, 7]. In the present case report, the patient was a chronic alcoholic. Usually, SpCC presents as exophytic, polypoid masses in the larynx [9], rarely as a flat lesion [10]. Similar gross features comprised of a polypoidal lesion on the tongue, which was rapidly grown in a period of one month, was reported in our case.

Histologically, in SpCC, the mesenchymal component typically forms the bulk of the tumour and the epithelial component often blends into it [4]. In the present study, the tumour was predominantly composed of spindle cells along with very few foci of malignant squamous cells. The spindle shape of the tumour cells has been considered to be caused by the lack of expression of cell adhesion molecule, such as cadherins and the consequent alteration of keratin filament network [2].

In the immunohistochemical study of our case, it was found that the spindle cell component was strongly positive for vimentin and focally for cytokeratin while cytokeratin positivity was shown by the squamous component. The vimentin positivity reflects that these bizarre fibroblast-like cells are carcinoma cells with true mesenchymal metaplasia. These results may explain that these cells have acquired mesenchymal properties both morphologically and functionally through metaplastic changes and simply correlated with the concept of a malignant epithelial cell undergoing alterations, resulting in a loss of keratin and acquiring vimentin as the cytoskeleton protein [2].

Three main theories have been proposed to explain the histogenetic nature of spindle cells. The first theory asserts that spindle cells and epithelial cells arise simultaneously from separate stem cells, deserving the name collision tumour. The second theory explains the nature of the spindle cell component as an atypical reactive proliferation of the stroma, and hence named pseudo sarcoma. Finally, according to the last theory, cells of both spindle and epithelial components have the same monoclonal origin, and dedifferentiation or transformation to spindle cells has occurred [4]. However, recently, monoclonal hypothesis is widely accepted and has been strongly supported by some studies [1, 4].

The differential diagnosis may be problematic in the cases where the main bulk of the tumour is formed by the spindle cell component. SpCC may imitate true fibrosarcoma but fibrosarcomas are rare in the head and neck regions. The presence of malignant squamous cells and immunohistochemical markers leads to a correct diagnosis. The spindle cell lesion also has to be differentiated from malignant fibrous histiocytoma (by its cytologic pleomorphism and multinucleate giant cell), rhabdomyosarcoma (by the presence of tadpole or strap cells), synovial sarcomas (by its age of presentation, location, and chromosomal translocation), and malignant peripheral nerve sheath tumours (showing nerve coursing of the tumour cells and herniation of tumour in blood vessels).

In this case, cytokeratin and vimentin positivity was more in favour of epithelial origin with squamous differentiation and mesenchymal participation in the genesis of SpCC [6]. The W.H.O. classification of tumours of the oral cavity and oropharynx has placed this entity under malignant epithelial tumours of SCC and labelled it “Spindle cell carcinoma” (SpCC) [2]. Disease progression in SpCC is reported to be characterised by recurrences and metastases [3]. Regional metastasis is more common than distant [4]. SpCC is more aggressive [2, 11] than the more usual SCC, and hence has lower overall survival [4]. Prognosis of SpCC has been related to the depth of invasion, polypoid exophytic growth pattern, a history of radiotherapy, vascular invasion, and the presence of regional and distant metastases [2, 7, 11].

The overall survival of SpCC is poorer than SCC [12]. Surgical resection with neck dissection is accepted as the best treatment of choice in the oral cavity [4, 7] and is aimed at controlling local and distant recurrence [11]. Surgical intervention, with or without radiotherapy, had better prognosis than radiotherapy alone. The role of chemotherapy in the treatment of SpCC is not explained. Our patient had undergone hemiglossectomy, and his condition was monitored with no recurrence to date.

## Conclusion

Thus, to summarise, though the tongue is a rare site for a spindle cell lesion, a differential diagnosis of SpCC in tumours of the head and neck region should always be mentioned if malignant spindle cells are seen, and a careful search should be performed for the epithelial component. Immunohistochemistry should be advised if there is even a small suspicion of epithelial component.
